# Identification of Autism Spectrum Disorder With Functional Graph Discriminative Network

**DOI:** 10.3389/fnins.2021.729937

**Published:** 2021-10-21

**Authors:** Jingcong Li, Fei Wang, Jiahui Pan, Zhenfu Wen

**Affiliations:** ^1^School of Software, South China Normal University, Guangzhou, China; ^2^Pazhou Lab, Guangzhou, China; ^3^Department of Psychiatry, New York University School of Medicine, New York, NY, United States

**Keywords:** autism spectrum disorder, ABIDE, graph neural network, functional graph, resting-state functional MRI

## Abstract

Autism spectrum disorder (ASD) is a specific brain disease that causes communication impairments and restricted interests. Functional connectivity analysis methodology is widely used in neuroscience research and shows much potential in discriminating ASD patients from healthy controls. However, due to heterogeneity of ASD patients, the performance of conventional functional connectivity classification methods is relatively poor. Graph neural network is an effective graph representation method to model structured data like functional connectivity. In this paper, we proposed a functional graph discriminative network (FGDN) for ASD classification. On the basis of pre-built graph templates, the proposed FGDN is able to effectively distinguish ASD patient from health controls. Moreover, we studied the size of training set for effective training, inter-site predictions, and discriminative brain regions. Discriminative brain regions were determined by the proposed model to investigate its applicability and biomarkers for ASD identification. For functional connectivity classification and analysis, FGDN is not only an effective tool for ASD identification but also a potential technique in neuroscience research.

## 1. Introduction

Autism spectrum disorder (ASD) is a specific brain disease that causes social and communication impairments, restricted interests, and repetitive behaviors (Lord et al., 2000; Yang et al., 2019). Early diagnosis of ASD is significant for preparing treatment plan and conducting early intervention for ASD. Human brain connectivity network aims to reveal structural and functional interactions between brain regions, which were proven to be potential in identifying predictive biomarkers for neurodevelopmental and neuropsychiatric disorders (Wu et al., 2006; Arslan et al., 2018).

The resting-state functional magnetic resonance imaging (rs-fMRI) is widely used to study functional connectivity between different brain regions with its high spatial resolution (Luca et al., 2006; Aggarwal et al., 2017). Based on rs-fMRI and functional connectivity, it is possible to develop a reliable and objective technique for early diagnosis of ASD (Abraham et al., 2017; Aggarwal and Gupta, 2019b; Dadi et al., 2019). To facilitate techniques for ASD identification with rs-fMRI data, a large multisite dataset termed Autism Brain Imaging Data Exchange (ABIDE) was released (Nielsen et al., 2013). The ABIDE dataset consists of the rs-fMRI data of ASD patients and healthy controls (HC) from different international acquisition sites and using different protocols. In the ABIDE dataset, the challenging problems in identifying ASD patients lie in the individual differences of functional connectivity as well as acquisition protocol differences.

In the past few years, many machine learning approaches were proposed for processing functional connectivity data and identifying ASD. As a popular machine learning method, support vector machine (SVM) was applied for ASD prediction based on Pearson's correlation functional connectivity (Nielsen et al., 2013). In another significant research on ABIDE dataset, the researchers investigated a few classifiers including random forests, Gaussian naive Bayes, support vector classifier, and ridge classifier (Abraham et al., 2017). The major contribution of this research lies in finding best predicting pipelines for ABIDE dataset, which were based on Multi Subject Dictionary Learning (MSDL) atlas, tangent space embedding, and ℓ_2_-regularized classifiers. Also evaluated on the ABIDE dataset, higher performance (e.g., accuracy over 75%) could be obtained by using extraneous information such as “multiple atlases” or “non-physiological” (Karampasi et al., 2020, 2021; Epalle et al., 2021). On the basis of correlation matrices computed from rs-fMRI time-series data, a probabilistic neural network was applied for ASD classification (Iidaka, 2015). A deep neural network based on stacked autoencoder was proposed to identify ASD patients from typical controls with T1-weighted MRI images that it outperformed some state-of-the-art methods (Kong et al., 2018). The deep learning technique also showed potential in identifying ASD based on behavior data like videos (Li et al., 2019). Compared with baseline methods, a mathematical framework based on Riemannian geometry and kernel methods achieved superior performance for functional connectivity graphs classification (Dodero et al., 2015).

Recently, graph representation methodology was proven to be a powerful tool in modeling structured data and achieved significant performance in many applications (Linial et al., 1995; Even, 2011; Aggarwal and Gupta, 2019a). Functional connectivity between brain regions in rs-fMRI data can be considered as typical structured data (Stam et al., 2008). Graph representation approaches also achieved impressive performance in dealing with functional connectivity data (Bullmore and Bassett, 2011; Sporns, 2011; Parisot et al., 2018). Accordingly, graph-based brain network is able to uncover system-level changes of brain regions (Wang et al., 2010). To predict progress of patients with mild cognitive impairment to Alzheimer's disease using rs-fMRI, graph theory, and machine learning approach were utilized (Hojjati et al., 2017). A graph convolutional network termed MTGCN was proposed for learning multi-scale graph representations of brain functional connectivity analysis with rs-fMRI data (Yao et al., 2019). Graph-based network also showed its potentials in ASD diagnosis that a siamese graph convolutional neural network (s-GCN) was proposed and evaluated on ABIDE dataset (Ktena et al., 2018). A graph convolutional neural network was proposed to model phenotypic and demographic information of subjects, and achieved significant improvements in ASD classification accuracy on ABIDE dataset (Parisot et al., 2018). However, due to individual differences and the unknown patterns of functional connectivity, conventional graph-based approaches perform poorly in some functional connectivity classification problems.

In this paper, we propose a modified graph convolution network for ASD classification and conduct a series of experiments on the ABIDE dataset. The main contributions of this paper can be summarized as follows:

1. An FGDN is proposed for ASD prediction based on resting-state functional MRI.
2. The proposed FGDN is able to achieve a high ASD classification performance on the ABIDE dataset.
3. Inter-site predictions and discriminative brain regions were studied with the proposed model to investigate its applicability and biomarkers for ASD identification.

The remainder of this paper is organized as follows. The proposed FGDN is presented in section 2. In section 3, numerical ASD classification experiments on the ABIDE dataset are carried out. In addition, the performance of the benchmark methods and the proposed methods are presented and compared. A general discussion of the proposed model is presented in section 4. Conclusions of this paper are given in section 5.

## 2. Materials and Methods

The functional connectivity is based on the hypothesis that different regions of interest (ROIs) could capture relevant functional activities within the brain. The ROIs are defined by brain structural atlases, such as Automated Anatomical Labeling (AAL) (Tzourio-Mazoyer et al., 2002), Harvard Oxford (HO) (Desikan et al., 2006), and Massive Online Dictionary Learning (MODL) (Dadi et al., 2019). According to previous research (Ktena et al., 2018; Dadi et al., 2019), functional connectivity is a typical structured data, which is suitable to define on a graph.

### 2.1. Graph Construction

Generally, a graph model can be constructed as follows:


(1) $$\begin{array}{l} \begin{array}{l} G=\left(V,E,W\right)\\ V=\left\{v_{i}|i=1,\ldots ,N\right\}\\ E=\left\{e_{ij}|v_{i},v_{j}\in V\right\}\\ W=\left\{w_{ij}\right\} \end{array} \end{array}$$

where V denotes the set of nodes (totally *N* nodes) in graph G, E are connected edges between different nodes, and *W* ∈ ℝ^*N* × *N*^ is the adjacency matrix whose element *w*_*ij*_ denotes the weight of connection between *i*th node and *j*th node. The element *w*_*ij*_ of adjacency matrix *W* is usually determined by the distance function and k-nearest neighbor rule. Gaussian kernel function is a commonly used distance function as


(2) $$\begin{array}{l} w_{ij}=\{\begin{array}{cc} exp\left(-\frac{{\left[dist\left(v_{i},v_{j}\right)\right]}^{2}}{2\theta^{2}}\right) & dist\left(v_{i},v_{j}\right)\leq \tau \\ 0 & otherwise \end{array} \end{array}$$

where *dist*(*v*_*i*_, *v*_*j*_) denotes the distance between *i*th node and *j*th node, τ and θ are two fixed parameters that determined by K-nearest neighbor (KNN) method for extracting *k* nearest nodes in graph G (Ktena et al., 2018; Song et al., 2019).

As mentioned above, the functional connectivity is a typical structured data that could be transformed into a graph (Ktena et al., 2018; Parisot et al., 2018; Dadi et al., 2019). Usually, each row vector of functional connectivity matrix is considered as one nodes in the graph. To model the connections between different nodes of functional connectivity graph, we utilized a functional graph construction technique (Ktena et al., 2018). Accordingly, such kind of graph structure could reflect functional connection weights between different regions from neuroscientific view.

The mean functional connectivity matrix and the corresponding graph structure are calculated as follows:


(3) $$\begin{array}{l} {\bar{F}}_{c}=\frac{1}{N_{c}}\sum_{i=1}^{N_{c}}F_{ic}={\left[f_{1},\ldots ,f_{i}\ldots ,f_{N}\right]}^{T}\\ V_{{\bar{F}}_{c}}=\left\{f_{i}|i=1,\ldots ,N\right\}\\ T_{c}=\left\{ℰ,W\right\}=\text{KNN}(V_{{\bar{F}}_{c}}) \end{array}$$

where *F*_*ic*_ denotes functional connectivity matrix of the *i*th training rs-fMRI data, *N*_*c*_ is the number of training samples, and *c* denotes the category of ASD (*c* = 0) or HC (*c* = 1); the row vector *f*_*i*_ of ${\bar{F}}_{c}$ is considered as the *i*th node feature of $V_{{\bar{F}}_{c}}$; *T*_*c*_ (termed graph template) denotes the connected edges E and their weights *W* between different nodes, E and *W* are determined by KNN method and Equations (1) and (2) with node features $V_{{\bar{F}}_{c}}$. The output graph template could be considered as the typical connections between nodes of ASD or HC samples.

**Figure 1:**
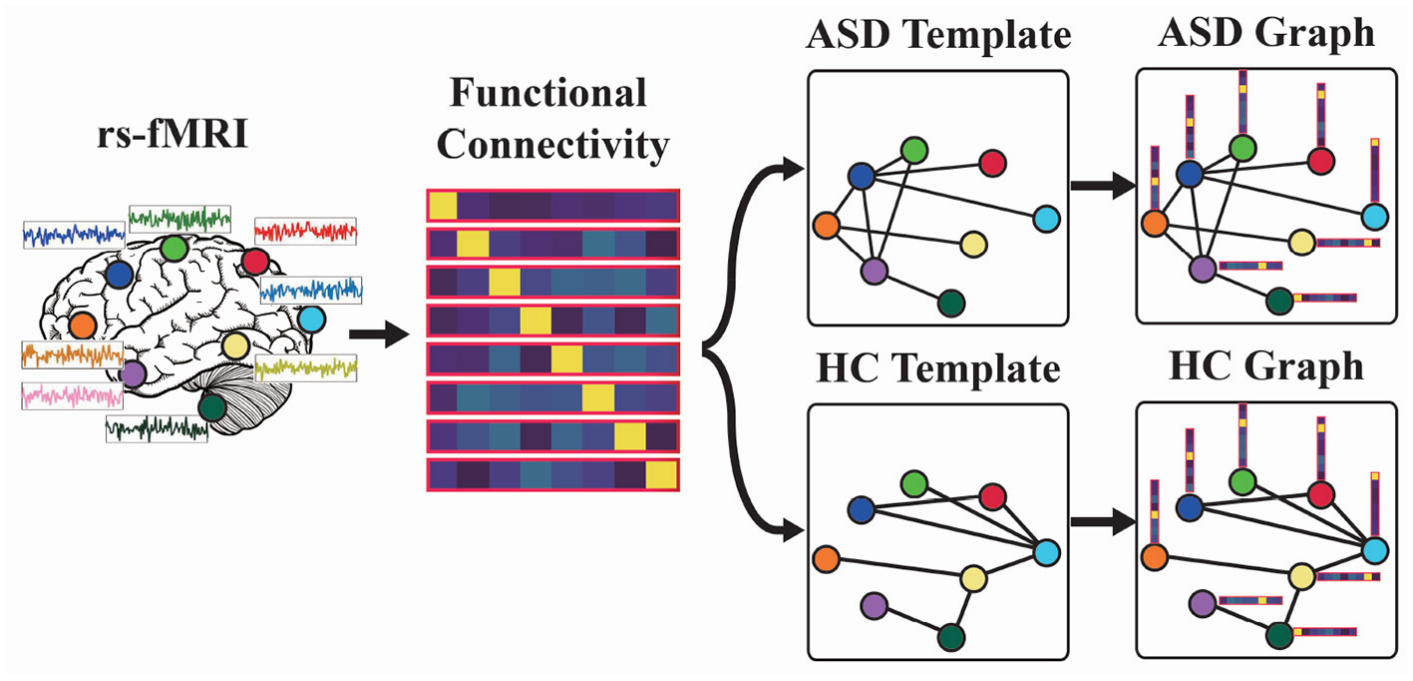
Functional graph construction: (1) Estimate functional connectivity matrix for each resting-state functional magnetic resonance imaging (rs-fMRI). (2) Build autism spectrum disorder/healthy controls (ASD/HC) template based on the mean functional connectivity matrix of ASD/HC samples. (3) Construct ASD and HC graphs.

Given the functional connectivity of a test rs-fMRI sample, it could be transformed into two graphs with the ASD and HC templates as shown in Figure 1. The interaction between the functional connectivity with the ASD/HC template is as follows:


(4) $$\begin{array}{l} F={\left[f_{1},\ldots ,f_{i}\ldots ,f_{N}\right]}^{T}\\ V_{F}=\left\{f_{i}|i=1,\ldots ,N\right\}\\ T_{c}=\left\{ℰ,W\right\}=\text{KNN}(V_{{\bar{F}}_{c}})\\ G_{c}=\left(V,ℰ,W\right)=\left(V_{F},T_{c}\right) \end{array}$$

where *F* is the input functional connectivity, the row vector *f*_*i*_ of **F** is the feature of the *i*th node in $V_{F}$, *T*_*c*_ is graph template mentioned in Equation (3), and $G_{c}$ is the output graph of ASD (*c* = 0) or HC (*c* = 1). For a test rs-fMRI sample, its output ASD graph and HC graph will have the same node features while the connections between their nodes are different.

As shown in Figure 1, we first estimate the functional connectivity of each rs-fMRI sample. Second, we will build the ASD/HC template based on the mean functional connectivity matrix of ASD/HC data according to Equation (3). Third, the functional connectivity will be combined with the ASD/HC template to construct ASD/HC graph. For a test rs-fMRI sample, we can obtain its node features (i.e., functional connectivity) while the connections between nodes are unknown. So we feed the node features into ASD graph template and HC template to construct ASD graph and HC graph, and then let the model to determine which graph matches the test sample. Therefore, we would like to build a model for ASD graph identification in the next section.

### 2.2. Proposed Graph Model

Generally, standard convolutional operations for regular data are inappropriate for processing graph data due to its irregular distribution of nodes (Krizhevsky et al., 2012). According to the previous research, two approaches can be utilized for generalizing convolution operations to graph data. First one is to rearrange all of the graph nodes into a regular grid and then conduct standard convolution operation (Niepert et al., 2016). However, the graph structure will be seriously corrupted in this way. The second approach is to apply spectral graph convolution (Bruna et al., 2013). By using convolutions in spectral domains with graph Fourier transform, spectral graph convolution can be feasibly applied on graph data. In addition, there are some following-up studies that the computational complexity of spectral graph convolution can be reduced from $O\left(n^{2}\right)$ to linear (Defferrard et al., 2016; Kipf and Welling, 2016).

According to previous research (Bruna et al., 2013; Yu et al., 2017; Song et al., 2019), spectral graph convolution is to multiply a signal *x* ∈ ℝ^*n*^ with a graph convolution kernel Θ and a graph convolution operator ${*}_{G}$ as,


(5) $$\begin{array}{l} \Theta {*}_{G}x=\Theta \left(L\right)x=\Theta \left(U\Lambda U^{T}\right)x=U\Theta \left(\Lambda \right)U^{T}x \end{array}$$

where graph Fourier basis *U* ∈ ℝ^*n* × *n*^ is the matrix of eigenvectors of the normalized graph Laplacian $L=I_{n}-D^{-1/2}WD^{-1/2}=U\Lambda U^{T}\in {\mathbb{R}}^{n\times n}$ (*I*_*n*_ is an identity matrix, *D* ∈ ℝ^*n* × *n*^ is the diagonal degree matrix with $D_{ii}=\underset{j}{\sum }W_{ij}$, *W* ∈ ℝ^*N* × *N*^ is the adjacency matrix mentioned in equation (1)); Λ ∈ ℝ^*n* × *n*^ is the diagonal matrix of eigenvalues of *L*, and filter Θ(Λ) is also a diagonal matrix. By this definition, a graph signal *x* is filtered by a kernel Θ with multiplication between Θ and graph Fourier transform *U*^*T*^*x* (Shuman et al., 2013). The computational complexity of spectral graph convolution is expensive due to $O\left(n^{2}\right)$ multiplications with graph Fourier basis.

In order to reduce computational complexity of spectral graph convolution, Chebyshev polynomials approximation technique was proposed (Defferrard et al., 2016). To reduce the number of parameters and localize the graph filter, the graph convolutional kernel Θ is restricted to a Chebyshev polynomial form as


(6) $$\begin{array}{l} \Theta \left(\Lambda \right)=\overset{K-1}{\underset{k=0}{\sum }}\theta_{\text{k}}T_{k}\left(\tilde{\Lambda }\right) \end{array}$$

where θ ∈ ℝ^*K*^ is the Chebyshev polynomial coefficients, *K* is the size of graph convolutional kernel which determines maximum convolutional range from central nodes, Λ is rescaled by $\tilde{\Lambda }=2\Lambda /\lambda_{\max}-I_{n}$ (λ_max_ denotes the largest eigenvalue of *L*, and *I*_*n*_ is a *N* × *N* identity matrix). The *T*_*k*_(*x*) could be recursively calculated as


(7) $$\begin{array}{l} \begin{array}{l} T_{0}\left(x\right)=1,T_{1}\left(x\right)=x\\ T_{\text{k}}\left(x\right)=2xT_{k-1}\left(x\right)-T_{k-2}\left(x\right),k\geq 2 \end{array} \end{array}$$

Then, the spectral graph convolution is rewritten as


(8) $$\begin{array}{l} \Theta {*}_{G}x=\Theta \left(L\right)x\;\approx \overset{K-1}{\underset{k=0}{\sum }}\theta_{\text{k}}T_{k}\left(\tilde{L}\right)x \end{array}$$

where $T_{\text{k}}\left(\tilde{L}\right)\in {\mathbb{R}}^{n\times n}$ is *k*-order Chebyshev polynomial estimation for the rescaled Laplacian $\tilde{L}=2L/\lambda_{\max}-I_{n}$. Then the computational complexity of spectral graph convolution by Equation (5) is reduced from $O\left(n^{2}\right)$ to O(K|ε|) (Defferrard et al., 2016).

**Figure 2:**
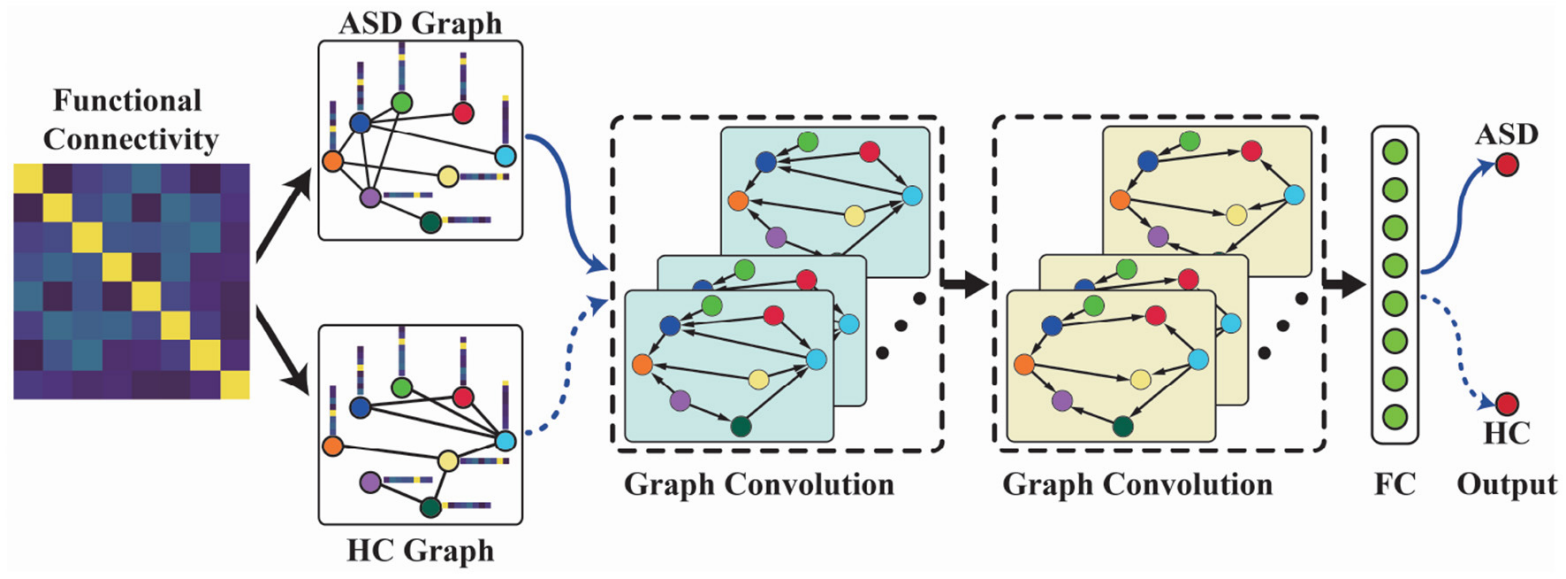
The functional graph discriminative network (FGDN) model for autism spectrum disorder (ASD) classification. The model consists of five layers, i.e., the functional graph construction layer, two graph convolutional layers, fully connected layer, and the output layer.

On the basis of functional graph construction and spectral graph convolution techniques, we proposed an FGDN for ASD classification. The framework of the proposed FGDN model is illustrated in Figure 2, which consists of five layers, i.e., the functional graph construction layer, two graph convolutional layers, fully connected layer, and one output layer. Given the ASD graph of an input sample, the corresponding output of the ASD unit (ASD output) is obtained. Likewise, the corresponding HC output is obtained by the HC graph of the same input sample. If the ASD output value is larger than the HC output, the input sample will be categorized as ASD. If not, it will be considered as HC class.

In the first layer of FGDN, each input functional connectivity data is transformed into ASD and HC graphs by Equations (1)–(5) and Figure 1. The number of graph nodes is determined by ROIs of the atlas, which were applied to model functional brain regions. In the paper, we will investigate the atlas of AAL (Tzourio-Mazoyer et al., 2002), Harvard Oxford (Desikan et al., 2006), and MODL (Dadi et al., 2019), which consists of 116, 118, or 128 ROIs (nodes). Then, the ASD graph and HC graph will be fed into the following network to obtain ASD and HC output, respectively. In each spectral graph convolution layer, there are 64 graph convolutional kernels. Moreover, we utilized *k*order Chebyshev spectral graph convolutional operation and applied *k* = 3 following the previous research (Ktena et al., 2018; Parisot et al., 2018). With two concatenated graph convolution layers, the graph features could be extracted hierarchically that is similar to convolution operations on grid data. The output of graph convolution layer is flatten to obtain the full connection (FC) layer. Parametric rectified linear unit (PReLU) activation function is applied in the two graph convolution layers as well as FC layer. Fully connected to the FC layer, the output layer of FGDN model consists of two units, i.e., the ASD and HC output units with sigmoid activation function.

Then, the proposed model can be trained by minimizing cross-entropy error of its predictions and the ground truth. As a result, the loss function is defined as


(9) $$\begin{array}{l} L=-\underset{i\in \Omega }{\sum }\underset{c}{\sum }y_{ic}\log\left(p_{ic}\right)+\left(1-y_{ic}\right)\log\left(1-p_{ic}\right) \end{array}$$

where *p*_*ic*_ is the value of ASD output unit (*c* = 1) or HC output unit (*c* = 2) of FGDN model with the input of the *i*th training sample, *p*_*ic*_ can be considered as the model's predicted probability of ASD or HC class, *y*_*ic*_ is the corresponding ground truth, and Ω denotes all of training samples.

In the next section, a series of experiments will be carried out to evaluate the proposed FGDN model. In addition, the corresponding experimental results of our method will be presented and compared with the other methods.

## 3. Results

In this section, the experimental procedure and results of the proposed FGDN will be presented. In the experiments, the proposed method and some other popular ASD classification methods are evaluated by the common ABIDE dataset. For ease of reproduction, the training procedure and the parameter settings of the proposed model are illustrated in the paper.

In our experiments, the hardware and software configuration of our system is a platform with Nvidia Titan Xp, Ubuntu 16.04, and Pytorch 1.2.0. The graph convolution operation of our work are achieved under the support of Pytorch-geometric 1.3.2 (Fey and Lenssen, 2019).

### 3.1. ABIDE Data Preprocessing

The ABIDE dataset was built for investigating the neural basis of autism and facilitating the development of ASD diagnosis techniques (Martino et al., 2014). We use the ABIDE data that were preprocessed by the previous researchers (Dadi et al., 2019) to discriminate individuals with autism spectrum disorder from healthy controls. In the preprocessed ABIDE dataset, there are 402 ASD samples and 464 HC samples. Following the pipeline of previous research (Dadi et al., 2019), we utilized Ledoit–Wolf regularized shrinkage estimator (Ledoit and Wolf, 2004) to efficiently estimate functional connectivity. According to previous research (Dadi et al., 2019), the tangent space embedding technique for estimating functional connectivity could effectively improve ASD classification performance. Likewise, we utilized tangent space embedding method to calculate the functional connectivity of each rs-fMRI samples.

Following the above instructions in Equations (1)–(5), ASD and HC functional graphs could be obtained. Here, the number of neighbors in KNN method was set to 20 for graph construction. Consequently, functional connectivity of each rs-fMRI data is transformed to be an ASD graph and an HC graph with the same node signals, i.e., the row vectors of its functional connectivity matrix. Each rs-fMRI signal will be transformed into ASD and HC graphs, then fed into the FGDN model and obtained the output values of ASD and HC units. Then the category of the input rs-fMRI sample could be identified.

### 3.2. Experiments

Before the experiment, the ABIDE dataset with 866 samples (402 ASD and 464 HC) is randomly split into five- or ten-folds for cross-validation (CV). Note that five- and ten-fold cross-validations were widely used in the previous studies. To evaluate the performance of different models, we measure the averaged CV accuracy and area under the curve (AUC) from the receiver operating characteristics curve.

In order to train the proposed FGDN model, Adam optimizer (Kingma and Ba, 2014) is applied for minimizing the model's loss function equation (9). The proposed model was trained by Adam optimizer with a learning rate of 0.0001, a weight-decay rate of 0.0005, and mini-batch size of 16. Drop-out operation with a rate of 0.1 was applied in the training procedure for randomly blocking the output units of graph convolution layers. Before the training procedure, we separated 10% training samples as monitoring set. Once the model achieved the highest accuracy on monitoring set, the training procedure was stopped to avoid overfitting. Finally, the trained FGDN model could be applied for ASD prediction.

Once the proposed FGDN model was trained, we could evaluate it and compare with the other models. As shown in Tables 1, 2, the experimental results of the proposed FGDN and many other methods on the ABIDE dataset. For evaluation, k-fold cross-validation strategy denoted as CV-n (*n* = 5 and 10) was applied. The averaged accuracy (ACC) of CV-5 and CV-10 along with standard deviation (SD) were presented. The corresponding area under the receive operating characteristic curve (AUC) results were also present. The used atlases, features, and number of subjects of different methods were present for analysis.

**Table 1:** Five-fold cross-validation performance.

**Method**	**Atlas**	**Feature**	**Number of subjects**	**ACC(SD)%**	**AUC(SD)%**
GaussianNB	MODL	Tangent	866	63.5 (3.1)	65.7 (5.4)
RandomF	MODL	Tangent	866	61.3 (1.8)	66.3 (4.7)
Logistic-L2	MODL	Tangent	866	64.1 (0.9)	69.8 (3.4)
Ridge	MODL	Tangent	866	63.8 (4.4)	63.4 (5.6)
KNN	MODL	Tangent	866	65.9 (4.6)	68.1 (5.2)
SVM-L1	None	Voxel	1,112	62.0 (-)	- (-)
sGCN	HO	Correlation	871	69.0 (-)	- (-)
ASD-SAENet	CC200	Correlation	1,035	70.8 (-)	- (-)
FGDN	HO	Correlation	866	65.7 (2.2)	67.8 (3.5)
FGDN	HO	Tangent	866	71.4 (4.2)	76.9 (3.6)
FGDN	AAL	Correlation	866	66.7 (5.2)	67.5 (3.8)
FGDN	AAL	Tangent	866	72.3 (3.0)	75.1 (4.5)
FGDN	MODL	Correlation	866	68.8 (2.6)	72.2 (1.9)
FGDN	MODL	Tangent	866	**72.5 (5.3)**	**77.8 (4.5)**

*The bold values indicate the largest values*.

**Table 2:** Ten-fold cross-validation performance.

**Method**	**Atlas**	**Feature**	**Number of subjects**	**ACC(SD)%**	**AUC(SD)%**
GaussianNB	MODL	Tangent	866	64.0 (5.7)	65.0 (6.7)
RandomF	MODL	Tangent	866	62.8 (3.3)	64.9 (5.5)
Logistic-L2	MODL	Tangent	866	64.8 (3.6)	69.0 (5.3)
ridge	MODL	Tangent	866	67.9 (3.2)	70.0 (5.2)
KNN	MODL	Tangent	866	65.8 (6.2)	66.6 (5.9)
SVC	MSDL	Tangent	871	66.9 (2.7)	- (-)
LSTM	CC200	Time Series	1,112	68.5 (5.5)	- (-)
InvNet	CC200	Correlation	1,035	71.0 (-)	- (-)
DNN	CC200	Correlation	1,035	70.0 (-)	- (-)
AttentionET	CC200	Correlation	1,054	**72.2 (-)**	- (-)
GCN	HO	Correlation	871	70.4 (3.9)	75.0 (4.6)
FGDN	HO	Correlation	866	67.9 (5.5)	69.5 (6.4)
FGDN	HO	Tangent	866	71.4 (4.3)	77.7 (5.3)
FGDN	AAL	Correlation	866	65.9 (3.5)	71.8 (5.1)
FGDN	AAL	Tangent	866	70.8 (5.2)	**79.4 (5.0)**
FGDN	MODL	Correlation	866	68.0 (3.4)	69.8 (5.2)
FGDN	MODL	Tangent	866	71.8 (4.5)	76.3 (5.6)

*The bold values indicate the largest values*.

Conventional classifiers like logistic regression with L2 regularization (Logistic-ℓ_2_) and support vector machine with ℓ_1_ regularization (SVM-ℓ_1_) were applied as benchmark methods for ASD classification (Rane et al., 2017; Dadi et al., 2019). The corresponding accuracies were 64.1(0.9) and 62.0(−) (− indicated an unknown score), respectively. The Logistic-ℓ_2_ was based on tangent feature of MODL atlas, while the SVM-ℓ_1_ was based on raw voxel values of standard anatomical space (MNI152). Support vector classifier (SVC) was applied to model tangent feature on MSDL atlas that ACC of 66.9(2.7) was achieved (Abraham et al., 2017). The baseline classifiers such as Gaussian Naive Bayes (GaussianNB), Random Forest (RandomF), Ridge regression (Ridge), and K-nearest neighbors (KNN) were also evaluated in the experiments.

Deep neural networks (DNN) achieved impressive performance in many areas. For ASD classification, a recurrent neural networks with long short-term memory (LSTM) was proposed to model time series on Craddock 200 (CC200) atlas and achieved ACC of 68.5(5.5) (Dvornek et al., 2017). With an ASD classification accuracy of 71.0, the invertible network (InvNet) was proven to be both effective at classification and finding interpretable biomarkers for ASD (Zhuang et al., 2019). As a popular technique in many areas, a DNN model was applied for ASD classification and achieved an ACC of 70.0 (Heinsfeld et al., 2018).

Recently, some studies indicated that graph convolutional network could effectively deal with structured data like functional connectivity. A siamese graph convolutional neural network (sGCN) was proposed for functional connectivity classification, which utilized Harvard Oxford (HO) atlas and Pearson correlation features (Ktena et al., 2018). The sGCN was evaluated by CV-5 on 871 ABIDE subjects and achieved an accuracy of 69%. Using the phenotypic information of subjects, a GCN method was proposed to model the connections between subjects that it achieved accuracy of 70.4(3.9) and AUC of 75.0(4.6) in CV-10 experiments (Parisot et al., 2018). An attention mechanism based on Extra-Trees algorithm (AttentionET) achieved an ACC of 72.2, which was state-of-the-art performance on CV-10 of ABIDE dataset (Liu et al., 2020). Based on sparse autoencoder, the ASD-SAENet achieved an accuracy of 70.8 on CV-5 of 1,035 subjects in ABIDE dataset (Almuqhim and Saeed, 2021).

As shown in Tables 1, 2, each baseline method had two validation setups, i.e., five-fold CV and 10-fold CV. The FGDN reached a high performance with ACC of 72.5(5.3) and AUC of 77.8(4.5) by five-fold cross-validation (CV-5), and ACC of 71.8(4.5) and AUC of 76.3(5.6) by 10-fold cross-validation (CV-10). The corresponding sensitivity and specificity were 73.9(4.7) and 65.2(5.4) on CV-5, and 74.6(7.0) and 67.8(7.7) on CV-10. Compared to the AttentionET method with sensitivity of 68.8 and specificity of 75.4, the proposed FGDN model had higher sensitivity and lower specificity in CV-10 experiments.

In Tables 1, 2, many baseline methods and the proposed FGDN model were evaluated on the same data subsets (866 samples) and the same input representations (AAL, HO, and MODL atlases with correlation and tangent features). The proposed FGDN achieved higher performance than the GaussianNB, RandomF, Ridge, and KNN. But some of the referenced methods used different atlases, different features, and different number of subjects that we could not compare them in a quantitative way. According to these results, the high performance of the proposed FGDN may depend on the MODL atlas and the tangent features. The combination of MODL and tangent feature was also proven to be most discriminative features on ASD classification according to the previous quantitative experiments (Dadi et al., 2019). Consequently, the cross-validation performance of FGDN demonstrated its effectiveness in ASD classification.

In the next section, we will discuss about the internal properties of the proposed model. It may be significant for further research.

## 4. Discussion

In this section, the proposed model will be discussed in details. According to the benchmark research on multiple publicly available rest-fMRI datasets (Dadi et al., 2019), the MODL and tangent features were proven to be the best features for rest-fMRI classification. As many previous research (Parisot et al., 2018; Dadi et al., 2019; Zhuang et al., 2019), the 10-fold cross-validation settings was widely used for experiments. Therefore, we all used MODL atlas and tangent features, and evaluated on the CV-10 experiments in the discussion section.

First of all, we present the loss-vs.-epochs curves of the models in the 10-fold CV experiments. In addition, the curves of accuracy and AUC scores were also present. Second, we discuss about the improved performance of FGDN model as the increasing size of training dataset. In the experiments, We varied the number of training samples while keeping a fixed-sized validation set. Third, we analyzed inter-site performance of FGDN model. Finally, the discriminative brain regions for ASD classification will be extracted for analysis.

**Figure 3:**
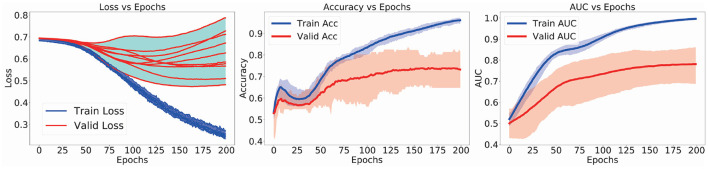
Loss(accuracy or AUC)-vs.-epochs curves in the 10-fold cross-validation (CV) training procedure.

In the left subfigure of Figure 3, the training(red)/validation(blue) loss-vs.-epochs curves of the 10 models in the CV-10 experiments are presented. During the training procedure, the model was trained to minimize its training loss. In the meanwhile, the validation loss were also decreased. Likewise, the training/validation curves of accuracy and AUC scores are present in the middle and right subfigures of Figure 3. The averaged training curve (red) and validation curve (blue) of accuracy/AUC in 10-fold CV experiments are presented. As the model was optimized by the training samples, the validation accuracy/AUC scores increased. In practice, we will separate 10% training samples as monitoring set to conduct early stopping and avoid overfitting. These curves demonstrated the effectiveness of the proposed method to model and discriminate ASD data.

**Figure 4:**
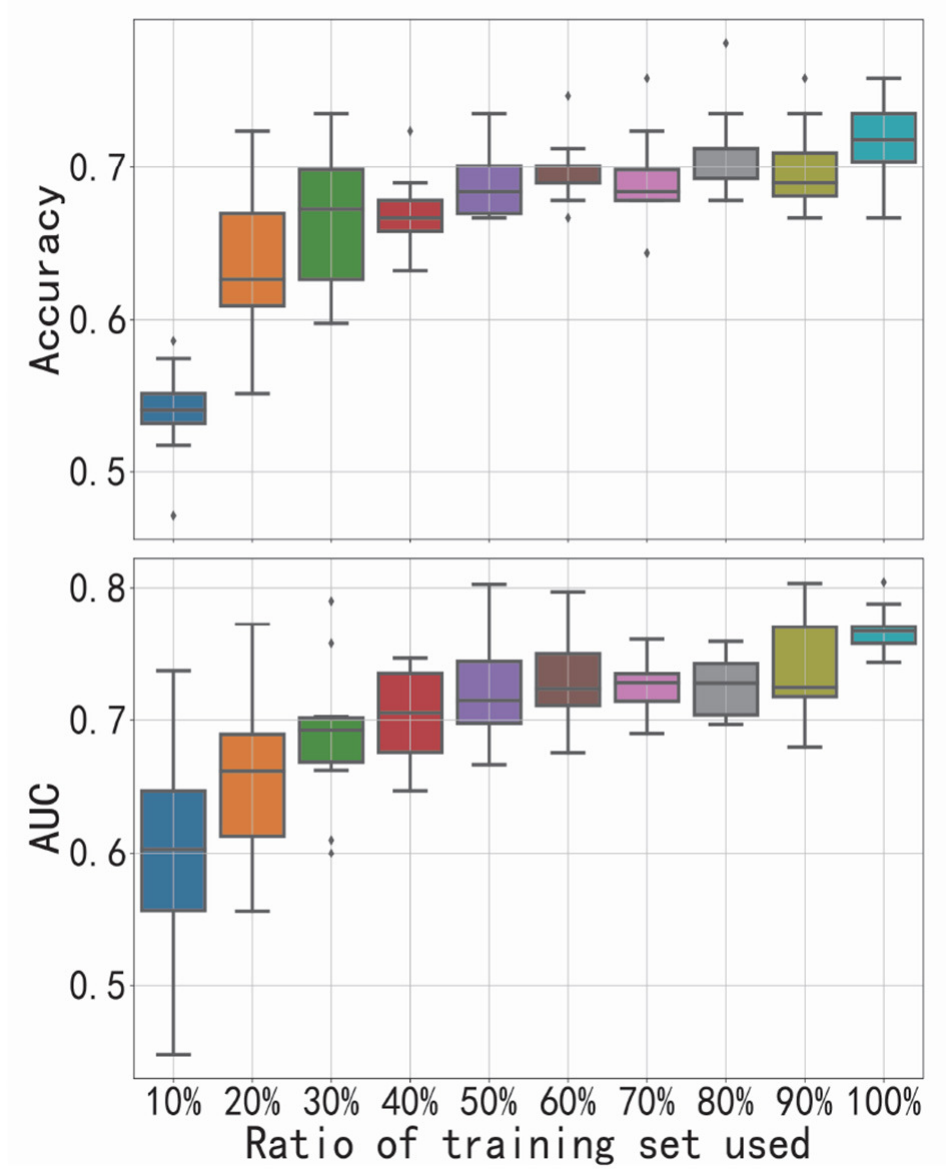
Performance of functional graph discriminative network (FGDN) as the increasing ratio of training set used. Boxplots denote the variant of classification accuracy/area under the curve (AUC) across 10-fold cross-validation.

As the increasing ratio of training set used, the corresponding performance of FGDN is presented in Figure 4. We changed the number of samples used for training the model (from 10 to 100% of the training set in CV-10), while the size of validation set (one-fold of CV-10) was unchanged. If the proposed model could work well with a relative small training set, it will be promising for application. With only 20% of training set, the model can achieve a performance better than chance level. With the increase in training samples, the accuracy or AUC also increases. The positive slope indicates that the addition of new training samples substantially improves performance.

Inter-site classification performance is also significant for the techniques in neuroscience (Dvornek et al., 2017; Dadi et al., 2019). Likewise, one of the challenging problems in identifying ASD patients lie in the individual differences of functional connectivity as well as acquisition protocol differences. In the experiment, leave-one-site-out strategy was applied that the samples of the studied site were used for testing while the samples of the rest sites were applied for training. And we used MODL atlas and tangent features for analysis. As shown in Table 3, we presented the performance of FGDN for ASD classification of all 20 sites. The proposed FGDN model achieved higher than chance level performance on each site with accuracies from 53.5 to 77.7, AUCs from 60.9 to 85.7. These results demonstrated the effectiveness of the proposed model to overcome different MRI facilities and different MRI settings for inter-site ASD classification.

**Table 3:** Leave-one-site-out autism spectrum disorder (ASD) classification performance of functional graph discriminative network (FGDN) on the Autism Brain Imaging Data Exchange (ABIDE) dataset.

**Site**	**ACC%/AUC%**	**Site**	**ACC%/AUC%**
NYU	65.8/67.5	CALTECH	73.0/70.3
UM_1	66.9/67.8	SDSU	77.7/81.3
USM	67.3/69.7	OLIN	75.6/71.9
UCLA_1	70.5/80.2	UM_2	67.3/65.0
PITT	70.5/68.5	LEUVEN_2	64.7/68.1
MAX_MUN	63.3/64.3	SBL	65.5/74.7
YALE	75.7/85.7	LEUVEN_1	53.5/60.9
KKI	75.8/84.5	OHSU	64.9/80.6
TRINITY	65.4/63.3	UCLA_2	76.4/83.0
STANFORD	68.6/79.3	CMU	60.5/79.7

**Figure 5:**
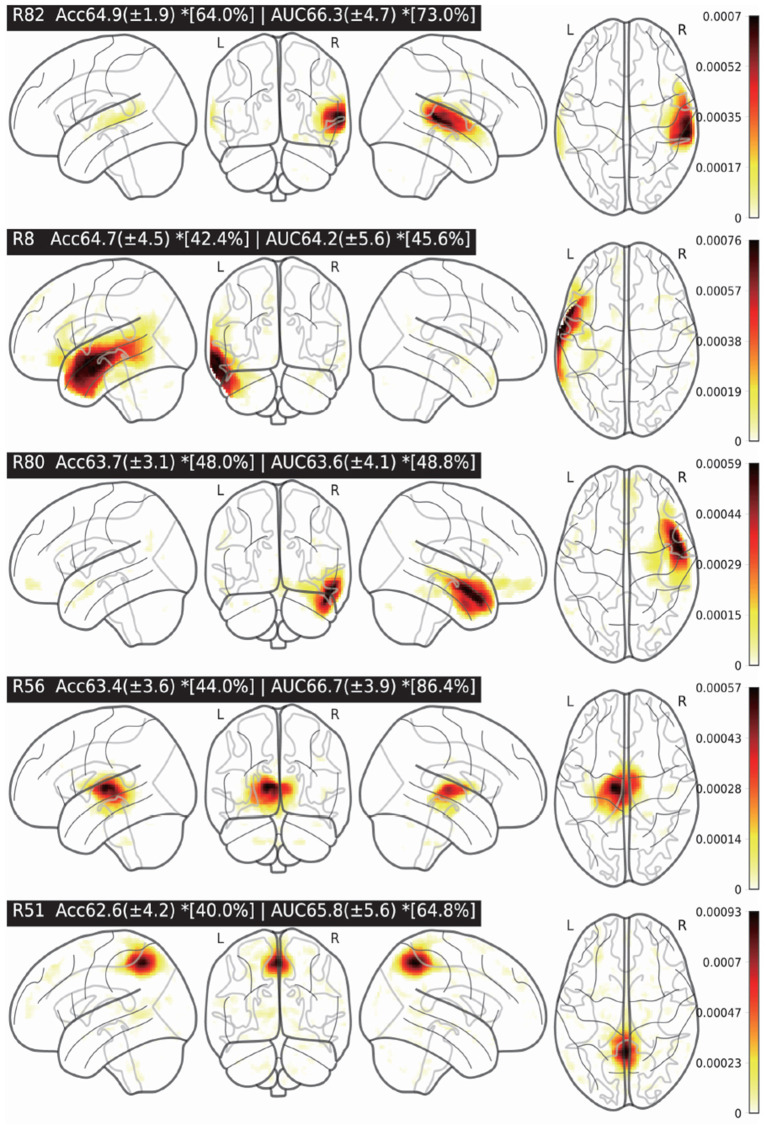
Five most discriminative brain regions and the classification averaged accuracy (ACC)/area under the curve (AUC) (±SD). The *[*x%*] of each region indicates that its ACC/AUC is significantly higher than that of the rest *x%* regions (*t*-test *p* < 0.05).

According to our experiments and the previous research (Dadi et al., 2019), the MODL atlas is promising for ASD classification performance. As a result, we would like to study the most discriminative brain region of MODL for ASD classification. In the MODL atlas, there are totally 128 regions that are represented by R *i*(*i* = 0…127) in the paper. In the experiments, only the features of the studied region will be kept unchanged while the features of the rest 127 regions were clamped to zeros. Then, we fine-tuned the model which were already trained in the previous CV-10 experiments. We considered that the averaged validation accuracy of each MODL region was its discriminative weight for ASD classification. As shown in Figure 5, the visualizations of the five most discriminative MODL regions (R82, R8, R80, R56, and R51) for ASD classification are presented. The classification AUC/ACC and the standard deviation (SD) of each region are presented as well. To study the differences in the discriminative power between the individual MODL regions, we applied *t*-test method to compare the ACC/AUC of different regions in CV-10 experiments. The *t*-test results of the five most discriminative brain regions are also presented in Figure 5. The *[*x%*] of each region indicates that its ACC/AUC is significantly higher than that of the rest *x%* regions with *t*-test *p* < 0.05. For example, the R82 region achieved an AUC of 66.3, which is significantly higher than the rest 73% brain regions. According to the R8, R80, and R82 regions, the superior temporal gyrus is the most discriminative region. This is consistent with findings from the neuroscience field that the cortical volume in several subregions of the superior temporal gyrus are abnormal in individuals with autism (Padmanabhan et al., 2017; Khosla et al., 2018; Zhuang et al., 2019). Therefore, it is a possible technique to determine the discriminative brain regions for ASD identification.

The above experiments and analysis of the proposed FGDN model are significant for studying the ASD. The proposed model can be considered as a new tool for ASD identification as well as the other neuroscience researches based on functional connectivity.

## 5. Conclusion

In this paper, an FGDN was proposed for identifying autism spectrum disorder. The FGDN model was built to discriminate functional graphs of ASD patients and health controls. The proposed model achieved a high ASD classification performance on the ABIDE dataset. In addition, inter-site predictions and discriminative brain regions for ASD prediction were investigated with the proposed model. The experiments and analysis demonstrated that the FGDN model is not only an effective model for identify autism patients but also a potential technique for neuroscience research. In the future, we would like to build more efficient networks to model functional connectivity and identify autism patients. Moreover, some new emerging machine learning techniques can also inspire the methodology in neuroscience like autism identification.

## Data Availability Statement

Publicly available datasets were analyzed in this study. This data can be found here: https://osf.io/hc4md/download.

## Ethics Statement

The studies involving human participants were reviewed and approved by Ethics Committee of South China Normal University. The patients/participants provided their written informed consent to participate in this study.

## Author Contributions

JL proposed the idea, conducted the experiments, and wrote the manuscript. FW provided advices on the research approaches and revised the manuscript. JP and ZW offered important help on guiding the experiments and analysis methods. All authors contributed to the article and approved the submitted version.

## Funding

This work was supported by the Key R&D Program of Guangdong Province under Grant 2018B030339001, the National Natural Science Foundation of China (Grant Nos. 62006082, 61836003, and 61906019), the Guangdong Natural Science Foundation (Grant Nos. 2021A1515011600, 2020A1515110294, and 2021A1515011853), and Guangzhou Science and Technology Plan Project (Grant No. 202102020877).

## Conflict of Interest

The authors declare that the research was conducted in the absence of any commercial or financial relationships that could be construed as a potential conflict of interest.

## Publisher's Note

All claims expressed in this article are solely those of the authors and do not necessarily represent those of their affiliated organizations, or those of the publisher, the editors and the reviewers. Any product that may be evaluated in this article, or claim that may be made by its manufacturer, is not guaranteed or endorsed by the publisher.
